# Perinatal outcome of immigrant women with and without refugee status compared to non-immigrant women: results of the pregnancy and obstetric care for refugees (PROREF) cross-sectional study

**DOI:** 10.1007/s00404-024-07639-3

**Published:** 2024-08-16

**Authors:** Darja Schlothauer, Louise Teschemacher, Jürgen Breckenkamp, Theda Borde, Matthias David, Michael Abou-Dakn, Wolfgang Henrich, Vera Seidel

**Affiliations:** 1 Klinik Für Geburtsmedizin, Charité Universitätsklinikum, Campus Virchow-Klinikum, Augustenburger Platz 1, 13353 Berlin, Germany; 2 Klinik für angeborene Herzfehler-Kinderkardiologie, Deutsches Herzzentrum Der Charité, Campus Virchow-klinikum, Augustenburger Platz 1, 13353 Berlin, Germany; 3 Fakultät Für Gesundheitswissenschaften, Arbeitsgruppe 3 Epidemiologie and International Public Health, Universität Bielefeld, Bielefeld, Germany; 4 Alice Salomon Hochschule Berlin, Alice-Salomon-Platz 5, 12627 Berlin, Germany; 5 Klinik Für Gynäkologie, Charité Universitätsklinikum, Campus Virchow-Klinikum, Augustenburger Platz 1, 13353 Berlin, Germany; 6 Klinik Für Gynäkologie Und Geburtshilfe, St. Joseph Krankenhaus, Wüsthoffstraße 15, 12101 Berlin, Germany

**Keywords:** Perinatal health, Refugee health, Immigrant health, Obstetric care, Maternal health, Migrant questionnaire, Social determinants of health

## Abstract

**Purpose:**

International studies show conflicting evidence regarding the perinatal outcome of immigrant women with and without refugee status compared to non-immigrant women. There are few studies about the situation in Germany. The research question of this article is: Is the perinatal outcome (Apgar, UApH (umbilical artery pH), NICU (neontatal intensive care unit) transfer, c-section rate, preterm birth, macrosomia, maternal anemia, higher degree perinatal tear, episiotomy, epidural anesthesia) associated with socio-demographic/clinical characteristics (migration status, language skills, household income, maternal education, parity, age, body mass index (BMI))?

**Methods:**

In the Pregnancy and Obstetric Care for Refugees (PROREF)-study (subproject of the research group PH-LENS), funded by the German Research Foundation (DFG), women giving birth in three centers of tertiary care in Berlin were interviewed with the modified Migrant Friendly Maternity Care Questionnaire between June 2020 and April 2022. The interview data was linked to the hospital charts. Data analysis was descriptive and logistic regression analysis was performed to find associations between perinatal outcomes and migration data.

**Results:**

During the research period 3420 women (247 with self-defined (sd) refugee status, 1356 immigrant women and 1817 non-immigrant women) were included. Immigrant women had a higher c-section rate (36.6% vs. 33.2% among non-immigrant women and 31.6% among women with sd refugee status, *p* = 0.0485). The migration status did not have an influence on the umbilical artery pH, the preterm delivery rate and the transfer of the neonate to the intensive care unit. Women with self-defined refugee status had a higher risk for anemia (31.9% vs. 26.3% immigrant women and 23.4% non-immigrant women, *p* = 0.0049) and were less often offered an epidural anesthesia for pain control during vaginal delivery (42.5% vs. 54% immigrant women and 52% non-immigrant women, *p* = 0.0091). In the multivariate analysis maternal education was explaining more than migration status.

**Conclusion:**

Generally, the quality of care for immigrant and non-immigrant women in Berlin seems high. The reasons for higher rate of delivery via c-section among immigrant women remain unclear. Regardless of their migration status women with low degree of education seem at increased risk for anemia.

**Supplementary Information:**

The online version contains supplementary material available at 10.1007/s00404-024-07639-3.

## What does this study add to the clinical work

**Table:** 

Immigrant women are at increased risk for delivery via c-section. Regardless of their migration status women with low degree of education are at increased risk for anemia.	

## Introduction

Migration is increasing worldwide. Some international studies show that the pregnancy outcome of immigrant women is worse compared to women from the corresponding host country, resulting in increased perinatal mortality. A survey from the Netherlands for the years 2000–2006 showed increased rates of fetal mortality depending on the country of origin. Compared to Dutch women, the OR for immigrant women from Africa was increased to 1.7 (95% CI 1,4–2,1), for immigrant women from South Asia to 1,8 (1,4–2,3), for other non-Western women to 1,3 (1,1–1,6) and for immigrant women from Turkey and Morocco to 1,3 (1,1–1,4) [1].

Other studies discuss the “healthy immigrant paradox”, especially in the USA and Canada. Several studies have shown a better perinatal outcome for first generation migrant women from Latin America compared to White American women, despite the poorer socioeconomic situation of Latinas [2–4].

A review of studies published between 1995 and 2008 showed conflicting evidence. Often studies showed less or as good numbers of preterm birth and low birthweight among migrants than receiving-country women. Only a meta-analysis distinguishing between region of origin identified immigrants at risk for poorer perinatal outcomes [5].

Refugee women are a subgroup of immigrant women. Due to the forced nature of their migration refugee women in general are at increased risk of adverse health outcomes [6]. During pregnancy socioeconomic and legal challenges are a potential additional burden for refugee women [7].

In Germany there is no nationwide survey on the pregnancy outcomes of immigrant women compared to non-immigrant women. The analysis of Berlin data for the period 1993–1999 showed that pregnant immigrants from Turkey appeared later for their first antenatal check-up and were more likely to suffer from anemia. Furthermore, these immigrant women were less likely to have received an epidural anesthesia (PDA) during childbirth to reduce pain [8].

For Berlin, neither between 1993 and 1999 nor in a later study between 2011 and 2012, there could be shown any relevant differences regarding preterm birth, neonatal Apgar values or severe neonatal acidosis depending on the migration status [8, 9].

A cross-sectional study conducted between 2010 and 2016 using administrative data of the main referral hospital for pregnant asylum seekers of the reception center of a large federal state in South Germany found lower odds ratios for high-risk pregnancy conditions (OR = 0.76, 95%CI 0.63–0.91, *p* < 0.0001), c-sections (OR = 0.84, 95%CI 0.66–1.07, *p* = 0.17) and perinatal complications (OR = 0.65, 95%CI 0.55–0.78, *p* < 0.0001) among asylum seeking women. The odds ratios for abortive outcomes/stillbirths (OR = 1.58, 95%CI 1.11–2.20, *p* = 0.01) and postnatal complications (OR = 1.80, 95%CI 0.93–3.19, *p* = 0.06) were higher among asylum seeking women relative to residents [10]. Furthermore, a recent study conducted in Germany, Austria, and Switzerland showed that immigrant women had lower knowledge about female health [11].

Often migrants are also struggling with economic problems [7] or some might have a lower degree of education, socioeconomic aspects also influence health-related behavior and potentially health outcomes. For Eastern Europe and Spain, it could be shown that women with lower socioeconomic status had worse perinatal outcomes [12–15] while for Scandinavia there was little effect of household income and maternal education on perinatal outcomes [16]. A recent systematic review of 46 studies showed that living in deprived areas increases the risk of adverse pregnancy outcomes [17].

In international comparison Germany has a net increase of immigrants. After outbreak of civil war in Syria, the percentage of immigrants in Germany rose by 8.5% between 2015 and 2016 [18]. Since 2022 due to conflict in Ukraine Germany is experiencing more immigration from Ukraine [19]. The increase of people seeking asylum in Germany since 2015 was the highest since the 1990s and challenged the German health care system [20].

The question of the study was: Is the perinatal outcome (Apgar, UApH (umbilical artery pH), NICU (neontatal intensive care unit) transfer, c-section rate, preterm birth, macrosomia, maternal anemia, higher degree perinatal tear, episiotomy, epidural anesthesia) associated with socio-demographic/clinical characteristics (migration status, language skills, household income, maternal education, parity, age, body mass index (BMI))?

Our hypothesis was that perinatal outcome of women with self-defined (sd) refugee status or immigrant women is worse than the perinatal outcome of non-immigrant women.

## Materials and methods

Collection of data: The Pregnancy and Obstetric Care for Refugees (PROREF)-study (subproject of the research group PH-LENS, funded by the German Research Foundation, funding labels DA 1199/5-1 and BO 3384/2-1) had the aim to examine the situation of obstetric care for women, who had fled their home country and reached Germany since the summer of 2015, compared to other immigrant women and non-immigrant women. The migration categories were not defined by German legal residence categories, but by self-information of the respective woman. Women who answered in the administered questionnaire that they had fled their home country and were in Germany since less than 5–7 years were grouped in the category “self-defined (sd) refugee status”. All other women being born outside of Germany were grouped as “immigrant” and all women being born in Germany were grouped as “non-immigrant”, irrespective of the migration status of their parents.

Between June 2020 and April 2022 data collection was performed in three hospitals of tertiary care in Berlin/Germany. Data collection was performed one to three days postpartum on the postpartum ward of the respective participating hospital by a team of female trained interviewers. All women of 18 years of age or older, who had given birth to one or more living babies in the delivery room of the respective participating hospital and speaking German, English, French, Arabic, Persian, Kurdish, Russian or Vietnamese could be included. Women who were in care of a caseload midwife were excluded from study participation, as continuity of care was suspected to be a potential confounder [21]. The women were informed about the study’s goals and modalities and after informed consent the interview was performed immediately afterward.

A modified and shortened version of the Migrant Friendly Maternity Care Questionnaire (MFMCQ) [22], which was evaluated in a pre-study, was used to assess migration specific data and the routine perinatal data from the hospital charts was linked to the migration specific data. The questionnaire was available in English, French, Arabic, German and Turkish [23, 24]. For the PROREF study a questionnaire version in Persian, Kurdish, Russian and Vietnamese was created.

The members of the interview team were trained in interview administration before data collection. They spoke German, English, French, Russian and/or Arabic. For women participating in Turkish, Persian, Kurdish or Vietnamese an in-person or telephone translator was consulted.

Data collection was planned on 7 days of the week. Due to personnel shortages or illnesses in the study team, occasionally days were not covered. The study was earlier described in detail by Seidel and Teschemacher et al. [25].

## Data analysis

Continuous variables were reported as mean or as median. Categorial variables were expressed as the number of participants and corresponding percentage. After descriptive analysis differences in characteristics were analyzed using Chi^2^ tests. The level of significance was defined as *p* < 0.05. Confounders were identified based on published evidence.

Primary outcome measures were low Apgar score at 5 min (Apgar5) (< 7), low pH of the umbilical artery (UApH) (≤ 7.1), transfer of the neonate to the neonatal intensive care unit (NICU), cesarean section (c-section), premature delivery (< 37 weeks + 0 weeks of pregnancy), birth weight > 4000 g, low maternal hemoglobin (hgb) value (< 10 g/dl), higher degree perineal tear (third or fourth degree), epidural anesthesia (EDA) and episiotomy.

We controlled for immigration group as defined above, maternal education, maternal age, parity, body mass index (BMI), household income and self-rated German language skills. Maternal education was grouped in (1) no or any education up to graduation from secondary school, (2) high-school diploma, (3) university education. Maternal age was grouped into four groups (18–24 years, 25–29 years, 30–34 years and > 34 years). Parity was dichotomized in primipara vs. multipara. BMI was grouped in four groups (< 18.5 kg/m^2^, ≥ 18.5 kg/m^2^ < 25.0 kg/m^2^, ≥ 25.0 kg/m^2^ < 30.0 kg/m^2^, ≥ 30.0 kg/m^2^). Household income was divided in five groups (< 900 €, 900–1500€, > 1500€–2600€, > 2600€–5000€, > 5000€). German language skills were self-rated and grouped in (1) native language, (2) good German proficiency and (3) poor German proficiency. The self-rated German language skills of women who answered to the respective question “very good” or “good” were subsumed as “good” and if women answered “sufficient”, “rather bad” or “none”, their language skills were categorized as “not good”.

All data analysis was performed using SAS 9.4.

The PROREF study was approved by the ethics committee of the Charité—Universitätsmedizin Berlin [EA1/316/1]. The guidelines by the Charité for good clinical practice and the requirements of the Berlin data protection act were followed.

## Results

### Study cohort

During the study period 18,636 deliveries occurred in the three study centers. Of the 17,555 women who fulfilled the inclusion criteria, 4413 women (25%) were invited to participate in the study. With 3420 interviews conducted and included in the analysis the response rate was high with 77.5%. In 42% of the interviews with women not born in Germany language interpretation was necessary (68.8% foreign language spoken by the interviewing study group member, 12.0% telephone interpreter, 10.3% interpretation via partner of the study participant). The study participants emigrated from 115 different countries of origin. The most frequent countries of origin were Syria (*N* = 157; 9.8%), Poland (*N* = 110; 6.9%), Vietnam (*N* = 84; 5.2%), Russia (*N* = 78; 4.9%) und Turkey (*N* = 69; 4.3%).

### Univariate analysis

Women with sd refugee status were younger (mean 29.05 years, range 18–43) than immigrant women (mean 32.76, range 18–51) or women born in Germany (mean 32.30, range 18–49). Different rates of underage pregnancies were not detected due to inclusion criterium of minimum age of 18 years. Women with sd refugee status were more frequently multipara and had a lower degree of formal education and a lower net household income. In the univariate analysis there was no difference in UApH, NICU transfer, premature delivery, birth weight or higher grade of perinatal tear between the three different groups of migration status.

Migration status was associated with the mode of delivery, the rate of maternal anemia and the option and usage of EDA in the univariate analysis. Immigrant women had a higher chance to deliver via c-section and a higher chance of an episiotomy in vaginal deliveries. Women with sd refugee status were at higher risk of anemia. Regarding EDA, women with sd refugee status were less often offered an EDA.

For an overview of socio-demographic and obstetric data see Table 1.

**Table 1:** Summary of socio-demographic and obstetric data (number of cases and/or %) according to migration group: sd refugee status, immigrant, non-immigrant

Migration status	1 Sd refugee status	2 Immigrant	3 Non-immigrant
N	233	1308	1758
Maternal education			
No or any education up to graduation from secondary school Total: 26.1%	102 (44.5%)	225 (17.4%)	526 (30.1%)
High-school diploma Total: 19.3%	66 (28.8%)	214 (16.5%)	351 (20.1%)
University education total: 54.7%	61 (26.6%)	856 (66.1%)	873 (49.9%)
Missings: *n* = 25	***p* (1 vs. 3) = < .0001**	***p* (2 vs. 3) = < .0001**	
Maternal age			
18–24 years of age	65 (27.9%)	84 (6.4%)	151 (8.6%)
25–29 years of age	61 (26.2%)	247 (18.9%)	338 (19.3%)
30–34 years of age	61 (26.2%)	510 (39.0%)	651 (37.1%)
35–51 years of age	46 (19.7%)	467 (35.7%)	615 (35.0%)
Missings: *n* = 3	*p*(1 vs. 3) = < .0001	*p*(2 vs. 3) = 0.1380	
Parity			
Primipara	89 (38.9%)	708 (54.9%)	1000 (57.9%)
Multipara	140 (61.1%)	581 (45.1%)	728 (42.1%)
Missings: *n* = 53	***p*(1 vs. 3) = < .0001**	*p*(2 vs. 3) = 0.1605	
BMI before pregnancy			
< 18.5 kg/m^2^	10 (4.4%)	81 (6.3%)	70 (4.1%)
≥ 18.5 kg/m^2^ < 25.0 kg/m^2^	97 (42.9%)	766 (59.8%)	1021 (59.7%)
≥ 25.0 kg/m^2^ < 30.0 kg/m^2^	64 (28.3%)	271 (21.2%)	357 (20.9%)
≥ 30.0 kg/m_2_	55 (24.3%)	162 (12.7%)	263 (15.4%)
Missings: *n* = 82	***p*(1 vs. 3) = < .0001**	***p*(2 vs. 3) = 0.0112**	
Household income			
< 900 €	68 (43.6%)	76 (6.5%)	51 (3.0%)
900–1500€	59 (37.8%)	132 (11.2%)	131 (7.8%)
> 1500€–2600€	24 (15.4%)	228 (19.4%)	310 (18.5%)
> 2600€–5000€	5 (3.2%)	457 (38.8%)	759 (45.3%)
> 5000€	0	284 (24.1%)	425 (25.4%)
Missings: *n* = 290	***p*(1 vs. 3) = < .0001**	***p*(2 vs. 3) = < .0001**	
Self-rated German skills (speaking, understanding and reading)			
Native language	0	84 (6.8%)	1615 (91.9%)
Good German proficiency	52 (25.4%)	672 (54.6%)	141 (8.0%)
Poor German proficiency	153 (74.6%)	476 (38.6%)	2 (0.1%)
Missings: *n* = 104	***p*(1 vs. 3) = < .0001**	***p*(2 vs. 3) = < .0001**	
APGAR5			
< = 6*	2 (0.9%)	25 (1.9%)	17 (1.0%)
> 6	231 (99.1%)	1273 (98.1%)	1736 (99.0%)
Missings: *n* = 15	*p*(1 vs. 3) = 0.8696	***p* (2 vs. 3) = 0.0250**	
UApH			
< = 7.1	6 (2.6%)	35 (2.7%)	65 (3.7%)
> 7.1	226 (97.4%)	1253 (97.3%)	1671 (96.3%)
Missings: *n* = 43	*p*(1 vs. 3) = 0.3743	*p*(2 vs. 3) = 0.1184	
NICU transfer			
Yes	14 (6.4%)	72 (5.7%)	96 (5.6%)
No	204 (93.6%)	1187 (94.3%)	1617 (94.4%)
Missings: *n* = 109	*p*(1 vs. 3) = 0.6236	*p*(2 vs. 3) = 0.8936	
Mode of delivery			
Vaginal	158 (68.4%)	819 (63.3%)	1159 (66.8%)
Cesarean section	73 (31.6%)	473 (36.6%)	575 (33.2%)
Missings: *n* = 42	*p*(1 vs. 3) = 0.6360	***p*(2 vs. 3) = 0.0485**	
Maternal hemoglobin			
< 8 g/dl	14 (6.3%)	52 (4.2%)	50 (3.0%)
< 10 g/dl	57 (25.6%)	277 (22.1%)	340 (20.4%)
> = 10 g/dl	152 (68.2%)	923 (73.7%)	1278 (76.6%)
Missings: *N* = 156	**1.vs 3 Chi2 *p* = 0.0049**	2 vs. 3 Chi2 *p* = 0.1025	
Offer EDA (in vaginal deliveries)			
No	88 (57.5%)	375 (46.0%)	555 (48.0%)
Yes, but rejected	10 (6.5%)	107 (13.1%)	173 (14.9%)
Yes, agreed	55 (36.0%)	334 (40.9%)	429 (37.1%)
Missings: *N* = 12	**1 vs. 3 Chi2 *p* = 0.0091**	2 vs. 3 Chi2 *p* = 0.1837	
Premature delivery			
< 34	3 (1.3%)	7 (0.5%)	17 (1.0%)
< 37	2 (0.9%)	43 (3.3%)	52 (3.0%)
> = 37 SSW	227 (97.8%)	1249 (96.2%)	1664 (96.0%)
Missings: *N* = 35	1 vs 3 Fishers exact test *p* = 0.1619	2 vs 3 Fishers exact test *p* = 0.2365	
Birth weight			
< 2.500 g	10 (4.3%)	53 (4.1%)	87 (5.0%)
2.500 g–4.000 g	198 (85.3%)	1118 (85.8%)	1466 (83.6%)
> 4.000 g	18 (7.8%)	109 (8.4%)	179 (10.2%)
> 4500 g	6 (2.6%)	23 (1.8%)	22 (1.3%)
Missings: *N* = 10	*p*(1 vs. 3) = 0.2542	*p*(2 vs. 3) = 0.1245	
Degree of perinatal tear			
No, I, II	154 (98.1%)	793 (97.8%)	1126 (97.7%)
IIIa, IIIb, IIIc, IV	3 (1.9%)	18 (2.2%)	26 (2.3%)
Missings: *N* = 16	*p*(1 vs. 3) = 0.7822	*p*(2 vs. 3) = 0.9560	
Episiotomy			
No	144 (92.9%)	708 (87.8%)	1038 (91.4%)
Yes	11 (7.1%)	98 (12.2%)	98 (8.6%)
Missing: *N* = 39	*p*(1 vs. 3) = 0.5204	***p*(2 vs. 3) = 0.0109**	

*P* value in bold indicates a statistically significant difference. Significance level was set at p < 0.05

Analysis was only performed on singleton births

*EDA* epidural anesthesia, *sd refugee status* self-defined refugee status, *UApH* umbilical artery pH

### Multivariate analysis

#### Maternal education

In the multivariate analysis of the perinatal outcomes the maternal degree of education is explaining more differences among groups than the migration status. The NICU transfer of the neonate was more likely if the mother had a low degree of education (7.7–8.3% vs. 3.7–5.1% for high degree of education, OR 1.93, *p* = 0.0046) (see Table 2). The Odds Ratio of a low Apgar at 5 min was highest for mothers with a low degree of education (OR 1.46), but this did not reach the level of significance (*p* = 0.3727). In the multivariate analysis regarding low Apgar values immigrant women had a higher chance (OR 2.85, *p* = 0.0349) of a low Apgar at 5 min than women with sd refugee status and non-immigrant women (see supplemental material: Table S1). The UApH was on the contrary neither influenced by the migration status nor the educational degree of the women. A higher maternal BMI was associated with a lower UApH (see supplemental material: Table S2).

**Table 2:** NICU transfer of the neonate

A absolute rates of NICU transfer by degree of maternal education			
	Low-school qualification	Middle-school graduation	High-school graduation
All women			
Transfer no	758 (92.3%)	580 (94.8%)	1647 (95.1%)
Transfer yes	63 (7.7%)	32 (5.2%)	85 (4.9%)
Missings: 143			
Sd refugee status			
Transfer no	88 (91.7%)	60 (93.8%)	52 (96.3%)
Transfer yes	8 (8.3%)	4 (6.3%)	2 (3.7%)
Missings: 19			
Immigrant			
Transfer no	198 (91.7%)	194 (95.1%)	784 (94.9%)
Transfer yes	18 (8.3%)	10 (4.9%)	42 (5.1%)
Missings: 66			
Non-immigrant			
Transfer no	472 (92.7%)	326 (94.8%)	8011 (95.2%)
Transfer yes	37 (7.3%)	18 (5.3%)	41 (4.8%)
Missings: 57			

b Chance of NICU transfer			
	Odds ratio	95% confidence interval	*p* value
Sd refugee status	1.65	0.71–3.85	0.2441
Immigrant	1.36	0.77–2.41	0.2949
Non-immigrant (Ref.)	1.00		
Primary school/no diploma and Secondary school diploma	1.93	1.22–3.03	**0.0046**
High-school diploma or comparable	1.16	0.70–1.91	0.5652
University degree (reference)	1.00		
18–24 years of age	1.00		
25–29 years of age	1.63	0.83–3.20	0.1579
30–34 years of age	1.10	0.55–2.18	0.7909
35 + years of age	1.51	0.75–3.05	0.2475
Primipara	1.00		
Multipara	0.67	0.46–0.98	**0.0400**
BMI < 18.5	0.67	0.27–1.64	0.3790
BMI < 25.0	1.00		
BMI < 30.0	0.72	0.46–1.13	0.1552
BMI > = 30.0	0.83	0.50–1.37	0.4621
Household income < 900€	0.76	0.35–1.68	0.7998
Household income 900–1500€	0.85	0.46–1.59	0.6183
Household income > 1500–2600€	0.84	0.50–1.42	0.5163
Household income > 2600–5000€	0.71	0.46–1.10	0.1220
Household income > 5000€	1.00		
Native speaker*	1.00		
Good German proficiency	0.76	0.42–1.38	0.3714
Bad German proficiency	0.86	0.44–1.69	0.6647
Birth mode vaginal delivery	1.00		
Birth mode primary cesarean section	1.00	0.56–1.79	0.9922
Birth mode secondary cesarean section	1.97	1.30–2.98	**0.0014**
Birth mode emergency cesarean section	8.31	3.41–20.25	** < .0001**
Birth mode vacuum extraction, forceps	1.75	1.02–3.01	0.0429
< 37 weeks of pregnancy	26.43	16.96–41.20	** < .0001**
> = 37 weeks of pregnancy	1.00		

Multivariate model *N* = 3102, events = 178

*NICU* neonatal intensive care unit

Women with a low degree of education were at higher risk (OR 1.46, *p* = 0.0014) for anemia. Furthermore, there was a tendency to more anemia with lower household income. Older maternal age and being multipara was associated with a lower chance of anemia (see Table 3). The rate of episiotomy was lower among women with lower degree of education (3.9–7.1% low educational level, 6.3–11.9% middle educational level, 5.0–14.2% high educational level) (see Table S3). There was a tendency to a higher chance to deliver via c-section with a lower degree of education (see Table 4).

**Table 3:** Chance for a low hemoglobin value (< 10 g/dl)

	Odds ratio	95% confidence interval	*p* value
Sd refugee status	1.10	0.73–1.66	0.6490
Immigrant	1.16	0.87–1.54	0.3102
Non-immigrant (Ref.)	1.00		
Primary school/no diploma and secondary-school diploma	1.47	1.17–1.85	**0.0011**
High-school diploma or comparable	1.33	1.05–1.67	**0.0174**
University degree (reference)	1.00		
18–24 years of age	1.00		
25–29 years of age	0.71	0.52–0.96	**0.0268**
30–34 years of age	0.63	0.46–0.86	**0.0032**
35 + years of age	0.62	0.45–0.86	**0.0037**
Primipara	1.00		
Multipara	0.64	0.54–0.77	** < .0001**
BMI < 18.5	0.94	0.64–1.38	0.7596
BMI < 25.0	1.00		
BMI < 30.0	1.03	0.84–1.28	0.7671
BMI > = 30.0	1.07	0.83–1.36	0.6084
Household income < 900€	1.31	0.90–1.90	0.1610
Household income 900–1500€	1.66	1.23–2.25	**0.0011**
Household income > 1500–2600€	1.05	0.81–1.37	0.7213
Household income > 2600–5000€	1.12	0.90–1.38	0.3076
Household income > 5000€	1.00		
Native speaker*	1.00		
Good german proficiency	1.12	0.84–1.49	0.4414
Bad german proficiency	1.15	0.82–1.60	0.4223

*N* = 3092, events = 781

**Table 4:** Mode of birth

	Sd refugee status	Immigrant	Non-immigrant
Vaginal delivery	137 (59.3%)	659 (51.0%)	967 (55.8%)
Primary cesarean section	33 (14.3%)	191 (14.8%)	231 (13.3%)
Secondary cesarean section	37 (16.0%)	267 (20.7%)	322 (18.6%)
Emergency cesarean section	3 (1.3%)	15 (1.2%)	22 (1.3%)
Vacuum extraction (+ Forceps*)	21 (9.1%)	160 (12.4%)	192 (11.1%)
Missings: *N* = 35	1 vs. 3 chi2 *p* = 0.7242	2 vs. 3 chi2 *p* = 0.1311	

The degree of maternal education did not influence the rate of preterm birth, the chance of a birthweight higher than 4 kg or the rate of higher degree perinatal tears (data not shown).

#### Mode of birth

Immigrant women had a higher chance to deliver via c-section than women with sd refugee status or non-immigrant women. Of the immigrant women 63.3% had a vaginal delivery, while non-immigrant women had a vaginal delivery in 66.6% and women with sd refugee status in 68.4% (see Table 1). The rate of primary and secondary c-sections was highest in immigrant women compared to non-immigrant women and women with sd refugee status. The rate of emergency c-sections was low and did not differ depending on migration status (see Table 4).

In the multivariate analysis the migration status remained significant as a factor increasing the chance to deliver via c-section. Furthermore, a higher maternal age and a higher BMI increased the chance of delivery via c-section. There was a tendency to a higher chance of delivery via c-section with lower degree of education. A moderately high income was associated with a lower chance of delivery via c-section. Speaking German not as a native language decreased the chances for a c-section (see Table 5).

**Table 5:** Chance of a cesarean section

	Odds ratio	95% confidence interval	*p* value
Sd refugee status	1.29	0.87–1.90	0.2136
Immigrant	1.64	1.26–2.14	**0.0002**
Non-immigrant (Ref.)	1.00		
Primary school/no diploma and secondary-school diploma	1.20	0.97–1.49	0.0898
High-school diploma or comparable	1.28	1.03–1.58	**0.0240**
University degree (reference)	1.00		
18–24 years of age	1.00		
25–29 years of age	1.30	0.93–1.82	0.1243
30–34 years of age	2.00	1.45–2.77	** < .0001**
35 + years of age	2.95	2.11–4.11	** < .0001**
Primipara	1.00		
Multipara	0.81	0.69–0.95	**0.0099**
BMI < 18.5	0.85	0.58–1.25	0.4093
BMI < 25.0	1.00		
BMI < 30.0	1.35	1.11–1.63	**0.0023**
BMI > = 30.0	2.14	1.72–2.67	** < .0001**
Household income < 900€	0.95	0.66–1.38	0.7902
Household income 900–1500€	1.00	0.74–1.34	0.9796
Household income > 1500–2600€	0.91	0.72–1.15	0.4322
Household income > 2600–5000€	0.78	0.65–0.94	**0.0088**
Household income > 5000€	1.00		
Native speaker	1.00		
Good german proficiency	0.67	0.52–0.88	**0.0036**
Bad german proficiency	0.70	0.51–0.95	**0.0228**

Vaginal delivery, vacuum extraction, forceps vs. primary cesarean section, secondary cesarean section, and emergency cesarean

*N* = 3206, events = 1099

To rule out that having given birth before in a different country influences the differences regarding the mode of birth depending on migration status, we performed a subgroup analysis of the mode of birth among primipara. In the univariate analysis there was a trend to a higher rate of c-sections among immigrant primipara. While 36.9% of immigrant primipara delivered via c-section only 26.1% of women with sd refugee status and 32.5% of non-immigrant women delivered via c-section (see Table 6a). In the multivariate analysis being immigrant was associated with a higher chance of a c-section for primipara (*p* = 0.0022) (see Table 6b). For primipara again it could be shown a trend toward lower chance of delivery via c-section if German was not spoken on the level of mother tongue. Also, the trend to higher chance of delivery via c-section with lower degree of education remained stable in this subgroup analysis (see Table 6b).

**Table 6:** Mode of birth depending on migration status among primipara

a Frequency of c-section depending on migration status among primipara			
	Sd refugee status	Immigrant	Non-immigrant
Cesarean section no	65 (73.9%)	442 (63.1%)	668 (67.5%)
Cesarean section yes	23 (26.1%)	258 (36.9%)	321 (32.5%)
	*p*(1 vs. 3) = 0.2230	*p*(1 vs.3) = 0.0605	

b Multivariate analysis: chance of delivery via c-Sectio, only primipara			
	Odds ratio	95% confidence interval	*p* value
Sd refugee status	1.21	0.65–2.25	0.5577
Immigrant	1.78	1.23–2.57	**0.0022**
Non-immigrant (Ref.)	1.00		
Primary school/no diploma and Secondary school diploma	1.58	1.16–2.15	**0.0038**
High-school diploma or comparable	1.22	0.91–1.64	0.1805
University degree	1.00		
18–24 years of age	1.00		
25–29 years of age	1.21	0.80–1.82	0.3599
30–34 years of age	2.20	1.46–3.30	**0.0001**
35 + years of age	2.80	1.84–4.25	** < .0001**
Primipara	0.65	0.39–1.08	0.0964
Multipara	1.00		
BMI < 18.5	1.58	1.21–2.07	**0.0008**
BMI < 25.0	2.12	1.54–2.93	** < .0001**
BMI < 30.0	0.88	0.51–1.52	0.6543
BMI > = 30.0	0.85	0.55–1.31	0.4539
Household income < 900€	1.03	0.75–1.42	0.8701
Household income 900–1500€	0.74	0.58–0.94	**0.0149**
Household income > 1500–2600€	1.00		
Household income > 2600–5000€	1.00		
Household income > 5000€	0.68	0.46–0.99	**0.0424**
Native speaker	0.74	0.48–1.14	0.1686

Vaginal delivery, vacuum extraction, forceps vs. primary cesarean section, secondary cesarean section, and emergency cesarean

*N* = 1777, events = 602

#### EDA during labor

As shown in Table 1 in the univariate analysis women with sd refugee status delivered more often without EDA. Women with sd refugee status in 57.5% of deliveries were not offered an EDA while 46.0% of immigrant and 48.0% of non-immigrant women were not offered an EDA (*p* = 0.0091) for pain control during delivery. Women with refugee status had the lowest rate of refusal against EDA (6.5%), if they were offered an epidural during labor (see Table 1).

In the multivariate analysis though, only being multipara remained significant as a factor lowering the chance of delivery with EDA (see Table S4a) and lowering the chance of being offered an EDA during labor (see Table S4b). Maternal educational level and language skills did not show an effect neither on chance of EDA nor on supply of EDA. An average monthly family income between 1500 and 2500 Euros was associated with higher chance of EDA during labor (see Table S4).

## Discussion

### Obstetric outcome

In general, in the PROREF cohort no clinically relevant outcome differences were observed depending on immigration status. The lower 5 min Apgar among immigrant women did not lead to higher degrees of transfer to the NICU. These results are in concordance with the previous studies by David et al., showing no serious differences in perinatal outcome depending on migration status for Berlin, Germany [8, 9]. Studies in other countries did not show such reassuring results. A US cohort study on 34,468,901 singleton births between 2009 and 2018 showed for example an increased risk for preterm delivery among non-Hispanic Native Hawaiian or other Pacific Islander immigrant women. This study on the other hand showed a higher risk for US-born women from other racially and ethnically minoritized groups than for their non-US-born counterparts [26]. In a Finnish study between 2004 and 2014, women from Sub-Sahara Africa, South and East Asia had a higher risk of preterm birth, while women from other parts of the world did not [27]. In Sweden between 1982 and 2006 there as an increased risk of preterm birth only among mothers from Eastern and Central Europa, Asia and Africa [28]. The PROREF cohort was too small to do subgroup analysis depending on country of origin. Furthermore, comparing the data to the results of Barreto et al. from the USA, only first-generation immigrants were categorized as immigrant women. Direct descendants of immigrant women were categorized as non-immigrant women.

A higher rate of anemia was found among women with sd refugee status. In the multivariate analysis though, maternal education was explaining more of these differences than the migration status itself. David et al. already detected a higher rate of anemia for immigrant women from Turkey in a study between 1993 and 1999 [8]. This might in part be explainable through a higher rate of inheritable forms of anemia among immigrant women [29, 30]. Nonetheless, in the multivariate analysis especially a low degree of maternal education and a low household income were associated with a higher rate of anemia, while the migration status lost the level of significance. Hence, in delivering care to pregnant women especially women of low social status should seem to be at risk for anemia. In case of anemia, they should be adequately counseled for a healthy diet and potentially also use of oral iron supplementation.

Regarding episiotomy some studies show a higher risk for immigrant women, for example in Austria [31]. The elevated risk for episiotomy in Australia among women from East Africa might be due to high prevalence of female genital cutting there and subsequent different obstetric measures during vaginal delivery [32]. The main countries of origin in the PROREF cohort were not East African. While in the univariate analysis we found a higher rate of episiotomy for immigrant women the migration status did not remain a significant factor explaining a higher chance of episiotomy in the multivariate analysis. Here a lower maternal education seems to be protective against an episiotomy.

Regarding the option of pain control with EDA, women with sd refugee status were offered an EDA least often. This association seems to be multifactorial, as in the multivariate analysis no clear explaining factor could be identified. In a previous study in Berlin, Germany, David et al. showed as well as a lower chance of delivery with EDA for women from Turkey, regardless of their reason for migration [8]. It could be shown for other countries before, that immigrant women less often had an EDA during labor [33, 34]. In a previous study on doctors and midwives in Berlin regarding the potential reasons for this, the potential role of language barrier leading to a lower rate of EDA among immigrant women was discussed. Doctors and midwives working in delivery rooms in Berlin had the impression that culturally an EDA might be less accepted, but also it might be offered less often in the context of a language barrier and potentially wrong translation via a family member might lead to a structural barrier to information about EDA and consecutively to a structural [35]. In a study by David et al. between 2011 and 2012 among immigrant women from Turkey there was no difference on EDA usage depending on migration status [36]. But in this study the EDA-rate was dependent on the German-proficiency of the women. Low German-proficiency was associated with a lower chance of EDA during labor (OR 0.67; 95% CI 0.48–0.95) [36]. In a study in Austria it could be shown as well that immigrant women had a higher rate of EDA if they were longer in Austria and had better German-proficiency [31]. On the contrary, in the PROREF study no influence of language proficiency neither on the offering nor on the usage of EDA could be observed.

### Mode of delivery

A study in Karlsruhe, Germany showed a lower cesarean section rate for women with refugee status between 2010 and 2016 compared to non-immigrant women [37]. A study in Taiwan on 673,830 singleton births between 2001 and 2003 showed a lower rate of delivery via c-section for immigrant mothers compared to Taiwan-born mothers [38]. Huang et al. did not differentiate the reason for migration among foreign-born mothers. In the PROREF study there was no difference in the cesarean section rate between women with refugee status and non-immigrant women. In a subgroup comparison of immigrants with and without refugee status, a higher cesarean section rate was demonstrated for immigrant women without sd refugee status. In an analysis from 2011 to 2012 for Berlin by David et al. there was no difference in the frequency of c-sections between immigrants, women with second or third generation migration background and women without migration background. However, this study showed that for the rare event of an emergency c-section (event overall: 1.58% of all births) an increased rate among immigrant women (18.4%) compared to non-immigrant women (15.1%) could be detected [39]. David et al. pointed out the great heterogeneity of cesarean section frequencies depending on the country of origin. The range of elective cesarean section rates ranged from 16.1% for immigrant women from India or other Asian countries to 7.2% for immigrants from the European Union. The rate of secondary cesarean sections in Berlin in 2011 and 2012 was lowest at 11.5% for immigrant women from Turkey and highest at 33.3% for women from North, Central and South Africa [39]. In the PROREF study presented here, no distinction was made according to type of cesarean section in the multivariate analysis. The higher rate of cesarean sections overall among immigrant women in the PROREF cohort in contrast to the survey by David et al. between 2011 and 2012 could be explained by a different composition of the countries of origin in the study group. David et al. overviewed mainly Turkey (24.9%) and Lebanon (13.5%) and only 5.4% were patients from other Arabic countries. In the PROREF cohort, most patients came from Syria (9.8%). Information about whether a c-section had already been performed in a previous pregnancy was not recorded in the PROREF study. To exclude a potential effect of a higher c-section rate in the country of origin, a subgroup analysis was performed for the mode of birth among primipara in the PROREF cohort. In the univariate analysis, there was only a tendency to more c-sections among immigrant women, but in the multivariate analysis again, being an immigrant women increased the chance of delivery via c-section. Thus, a higher rate of previous c-sections among immigrant women cannot explain their higher rate of c-sections alone. None the less, recommendations and practices from the countries of origins might shape the wishes and expectations of immigrant women in Germany. A study in Australia analyzing 237,943 Australian and 4057 Eastern African singleton births found higher odds for first-time birth via c-section for women born in Eastern Africa [40]. The authors suppose factors such as communication difficulties being a reason for this difference as socio-demographic and clinical risks did not explain it. In the PROREF study self-rated German language skills were assessed and interestingly speaking German less well seems to be protective against giving birth via c-section. The mechanism why the likelihood of a cesarean section is lower if a woman does not speak German as a native language remains unclear.

#### Strength and limitations of the study

*Strength*: (1) The PROREF-Studie is the biggest prospective quantitative cross-sectional study in Germany on the perinatal health of women with sd refugee status. (2) The response rate was high (77.5%). (3) Perinatal data could be linked to migration specific parameters. (4) Due to the extensive use of interpreters and the recruitment on the postpartum ward, women usually underrepresented in other studies could be included.

*Limitations*: (1) As data collection was only performed in centers of tertiary care in Germany’s capital Berlin, it remains questionable if the study sample can be regarded as representative. (2) Due to the wide range of countries of origin, it was statistically not sound to perform an analysis according to country of origin. (3) Women with a peripartal loss of a child were not included in the study sample, so the question of higher neonatal mortality remains unanswered.

*Conclusions and recommendations*: The quality of obstetric care in Berlin seems generally high for immigrant and non-immigrant women. Maternal education level seems to have a more important influence on perinatal outcomes than migration status. Interventions to improve perinatal health should be tailored to women with low level of formal education.

## Supplementary Information

Below is the link to the electronic supplementary material.

Supplementary file1 (DOCX 22 KB)

## Data Availability

The data set is available from the corresponding author on reasonable request.
