# Supplementary material for: Perinatal outcome of immigrant women with and without refugee status compared to non-immigrant women: results of the pregnancy and obstetric care for refugees (PROREF) cross-sectional study
Source: Arch Gynecol Obstet. 2024 Aug 16;310(5):2413–24. doi: 10.1007/s00404-024-07639-3 (PMC11485109; doi:10.1007/s00404-024-07639-3)
Supplement: Supplementary file 1 — Supplementary file1 (DOCX 22 KB) [file 404_2024_7639_MOESM1_ESM.docx]

| **Table S1: Chance of a low Apgar at 5 minutes** (logistic regression analysis – Apgar groups as above in descriptive table)  N=3233, events=44 | | | |
| --- | --- | --- | --- |
|  | Odds Ratio | 95 % confidence interval | p-value |
| Sd refugee status | 0.98 | 0.17 – 5.62 | 0.9821 |
| Immigrant | 2.85 | 1.08 – 7.54 | **0.0349** |
| Non-immigrant | 1.00 |  |  |
| Primary school / no diploma and Secondary school diploma | 1.46 | 0.64 – 3.32 | 0.3727 |
| High school diploma or comparable | 1.22 | 0.53 – 2.81 | 0.6407 |
| University degree (reference) | 1.00 |  |  |
| 18 – 24 years of age | 1.00 |  |  |
| 25 – 29 years of age | 1.36 | 0.35 – 5.28 | 0.6567 |
| 30 – 34 years of age | 1.60 | 0.43 – 6.00 | 0.4878 |
| 35+ years of age | 1.24 | 0.31 – 4.93 | 0.7599 |
| Primipara | 1.00 |  |  |
| Multipara | 1.02 | 0.54 – 1.93 | 0.9440 |
| BMI < 18.5 | 0.82 | 0.19 – 3.54 | 0.7941 |
| BMI < 25.0 | 1.00 |  |  |
| BMI < 30.0 | 0.83 | 0.37 – 1.86 | 0.6496 |
| BMI >=30.0 | 0.97 | 0.41 – 2.33 | 0.9529 |
| Household income <900 € | 2.56 | 0.75 – 8.71 | 0.1323 |
| Household income 900 – 1500 € | 1.01 | 0.26 – 3.940 | 0.9903 |
| Household income >1500 – 2600 € | 1.00 | 0.32 – 3.11 | 0.9971 |
| Household income >2600 – 5000 € | 2.09 | 0.94 – 4.65 | 0.0695 |
| Household income >5000 € | 1.00 |  |  |
| Native speaker* | 1.00 |  |  |
| Good german proficiency | 0.47 | 0.16 – 1.34 | 0.1555 |
| Bad german proficiency | 1.01 | 0.35 – 2.88 | 0.9853 |
| No variance inflation |  |  |  |

| **Table S2: Chance of a low UApH (logistic regression analysis) UApH groups: <=6 vs. >6**  N=3176, events=101 | | | |
| --- | --- | --- | --- |
|  | Odds Ratio | 95 % confidence interval | p-value |
| Sd refugee status | 1.09 | 0.37 – 3,25 | 0.8714 |
| Immigrant | 1.07 | 0.53 – 2.7 | 0.8536 |
| Non-immigrant (Ref.) | 1.00 |  |  |
| Primary school / no diploma and Secondary school diploma | 0.63 | 0.34 – 1.18 | 0.1491 |
| High school diploma or comparable | 0.83 | 0.46 – 1.49 | 0.5304 |
| University degree (reference) | 1.00 |  |  |
| 18 – 24 years of age | 1.00 |  |  |
| 25 – 29 years of age | 0.83 | 0.34 – 2.02 | 0.6867 |
| 30 – 34 years of age | 1.15 | 0.49 – 2.68 | 0.7505 |
| 35+ years of age | 1.00 | 0.41 – 2.43 | 0.9980 |
| Primipara | 1.00 |  |  |
| Multipara | 0.96 | 0.61 – 1.53 | 0.8659 |
| BMI < 18.5 | 1.22 | 0.47 – 3.16 | 0.6882 |
| BMI < 25.0 | 1.00 |  |  |
| BMI < 30.0 | 1.84 | 1.11 – 3.05 | **0.0180** |
| BMI >=30.0 | 2.47 | 1.39 – 4.38 | **0.0020** |
| Household income <900 € | 1.88 | 0.77 – 4.57 | 0.1623 |
| Household income 900 – 1500 € | 0.57 | 0.21 – 1.54 | 0.2676 |
| Household income >1500 – 2600 € | 0.67 | 0.34 – 1.32 | 0.2435 |
| Household income >2600 – 5000 € | 0.82 | 0.50 – 1.33 | 0.4191 |
| Household income >5000 € | 1.00 |  |  |
| Native speaker* | 1.00 |  |  |
| Good german proficiency | 0.64 | 0.31 – 1.34 | 0.2410 |
| Bad german proficiency | 0.43 | 0.18 – 1.04 | 0.0618 |
| Birth mode vaginal delivery | 1.00 |  |  |
| Birth mode primary cesarean section | 0.14 | 0.03 – 0.59 | **0.0071** |
| Birth mode secondary cesarean section | 0.81 | 0.45 – 1.47 | 0.4854 |
| Birth mode emergency cesarean section | 6.66 | 2.56 – 17.30 | **0.0001** |
| Birth mode vacuum extraction, forceps | 2.86 | 1.72 – 4.77 | **<.0001** |
| <37 weeks of pregnancy | 1.34 | 0.52 – 3.47 | 0.5477 |
| >=37 weeks of pregnancy | 1.00 |  |  |
|  |  |  |  |

| **Table S3: chance of episiotomy in vaginal delivery**  N=2068, events=207 | | | |
| --- | --- | --- | --- |
| Table S3a: chance of episitomy yes vs. no | | | |
|  | Odds Ratio | 95 % confidence interval | p-value |
| Sd refugee status | 1.13 | 0.48 – 2.62 | 0.7837 |
| Immigrant | 1.42 | 0.83 – 2.42 | 0.1967 |
| Non-immigrant (Ref.) | 1.00 |  |  |
| Primary school / no diploma and Secondary school diploma | 0.45 | 0.27– 0.75 | **0.0024** |
| High school diploma or comparable | 0.68 | 0.44 – 1.05 | 0.0841 |
| University degree (reference) | 1.00 |  |  |
| 18 – 24 years of age | 1.00 |  |  |
| 25 – 29 years of age | 0.73 | 0.41 – 1.36 | 0.3049 |
| 30 – 34 years of age | 0.79 | 0.44 – 1.43 | 0.4339 |
| 35+ years of age | 0.87 | 0.47 – 1.61 | 0.6548 |
| Primipara | 1.00 |  |  |
| Multipara | 0.26 | 0.18 – 0.38 | **<.0001** |
| BMI < 18.5 | 1.09 | 0.60 – 1.96 | 0.7810 |
| BMI < 25.0 | 1.00 |  |  |
| BMI < 30.0 | 0.78 | 0.51 – 1.19 | 0.2499 |
| BMI >=30.0 | 0.76 | 0.42 – 1.36 | 0.3519 |
| Household income <900 € | 0.67 | 0.27 – 1.65 | 0.3845 |
| Household income 900 – 1500 € | 1.63 | 0.93 – 2.84 | 0.0862 |
| Household income >1500 – 2600 € | 0.81 | 0.49 – 1.35 | 0.4241 |
| Household income >2600 – 5000 € | 1.09 | 0.77 – 1.56 | 0.6240 |
| Household income >5000 € | 1.00 |  |  |
| Native speaker* | 1.00 |  |  |
| Good german proficiency | 0.81 | 0.47 – 1.40 | 0.4587 |
| Bad german proficiency | 1.20 | 0.66 – 2.20 | 0.5457 |
|  |  |  |  |
| Table S3b |  |  |  |
| *Episiotomy depending on the education (vaginal delivery)* |  |  |  |
|  | Low school qualification | Middle school graduation | High school graduation |
| *Sd refugee status* |  |  |  |
| *No* | *65 (92.9 %)* | *38 (90.5 %)* | *38 (95.0 %)* |
| *yes* | *5 (7.1 %)* | *4 (9.5 %)* | *2 (5.0 %)* |
| *Missings:6* |  |  |  |
| *Immigrant* |  |  |  |
| *No* | *128 (94.8 %)* | *119 (88.2 %)* | *455 (85.9 %)* |
| *yes* | *7 (5.2 %)* | *16 (11.9 %)* | *75 (14.2 %)* |
| *Missings: 19* |  |  |  |
| *Non-immigrant* |  |  |  |
| *No* | *323 (96.1 %)* | *207 (93.7 %)* | *506 (87.9 %)* |
| *yes* | *13 (3.9 %)* | *15 (6.3 %)* | *71 (12.2 %)* |
| *Missings: 26* |  |  |  |

| **Table S4: Usage of EDA in vaginal deliveries**  **EDA:** epidural anesthesia | | | |
| --- | --- | --- | --- |
| **Table S4b:** Chance of delivering with EDA in vaginal deliveries  Yes, agreed versus yes, rejected and no  N=2097, events=811 | | | |
|  | Odds Ratio | 95 % confidence interval | p-value |
| Sd refugee status | 1.39 | 0.86 – 2.25 | 0.1830 |
| Immigrant | 1.18 | 0.86 – 1.63 | 0.3096 |
| Non-immigrant (Ref.) | 1.00 |  |  |
| Primary school / no diploma and Secondary school diploma | 0.80 | 0.61 – 1.04 | 0.0931 |
| High school diploma or comparable | 0.87 | 0.67 – 1.13 | 0.3030 |
| University degree | 1.00 |  |  |
| 18 – 24 years of age | 1.00 |  |  |
| 25 – 29 years of age | 1.01 | 0.71 – 1.44 | 0.9697 |
| 30 – 34 years of age | 1.15 | 0.80 – 1.64 | 0.4450 |
| 35+ years of age | 1.19 | 0.82 – 1.73 | 0.3659 |
| Primipara | 1.00 |  |  |
| Multipara | 0.30 | 0.25 – 0.37 | **<.0001** |
| BMI < 18.5 | 1.06 | 0.71 – 1.58 | 0.7842 |
| BMI < 25.0 | 1.00 |  |  |
| BMI < 30.0 | 0.95 | 0.74 – 1.21 | 0.6691 |
| BMI >=30.0 | 1.00 | 0.73 – 1.36 | 0.9810 |
| Household income <900 € | 1.11 | 0.70 – 1.76 | 0.6602 |
| Household income 900 – 1500 € | 1.16 | 0.80 – 1.67 | 0.4185 |
| Household income >1500 – 2600 € | 1.35 | 1.01 – 1.81 | **0.0432** |
| Household income >2600 – 5000 € | 1.16 | 0.92 – 1.47 | 0.1975 |
| Household income >5000 € | 1.00 |  |  |
| Native speaker | 1.00 |  |  |
| Good german proficiency | 1.11 | 0.81 – 1.53 | 0.5295 |
| Bad german proficiency | 0.94 | 0.64 – 1.38 | 0.7360 |
| **Table S4b:** Chance of being offered an EDA during vaginal delivery  Yes, agreed versus yes, rejected and no  N=2097, events=1099 | | | |
|  | Odds Ratio | 95 % confidence interval | p-value |
| Sd refugee status | 0.95 | 0.60 – 1.52 | 0.8316 |
| Immigrant | 1.22 | 0.89 – 1.67 | 0.2233 |
| Non-immigrant (Ref.) | 1.00 |  |  |
| Primary school / no diploma and Secondary school diploma | 0.98 | 0.76 – 1.26 | 0.8688 |
| High school diploma or comparable | 0.93 | 0.72 – 1.20 | 0.5755 |
| University degree | 1.00 |  |  |
| 18 – 24 years of age | 1.00 |  |  |
| 25 – 29 years of age | 0.96 | 0.68 – 1.36 | 0.8316 |
| 30 – 34 years of age | 0.92 | 0.65 – 1.31 | 0.6498 |
| 35+ years of age | 1.03 | 0.71 – 1.49 | 0.8739 |
| Primipara | 1.00 |  |  |
| Multipara | 0.33 | 0.27 – 0.40 | **<.0001** |
| BMI < 18.5 | 1.06 | 0.71 – 1.58 | 0.4731 |
| BMI < 25.0 | 1.00 |  |  |
| BMI < 30.0 | 0.88 | 0.70 – 1.11 | 0.2926 |
| BMI >=30.0 | 1.02 | 0.76 – 1.37 | 0.8977 |
| Household income <900 € | 1.22 | 0.78 – 1.89 | 0.3826 |
| Household income 900 – 1500 € | 1.05 | 0.74 – 1.49 | 0.7857 |
| Household income >1500 – 2600 € | 1.41 | 1.06 – 1.88 | **0.0173** |
| Household income >2600 – 5000 € | 1.20 | 0.96 – 1.50 | 0.1170 |
| Household income >5000 € | 1.00 |  |  |
| Native speaker | 1.00 |  |  |
| Good german proficiency | 1.01 | 0.74 – 1.37 | 0.9762 |
| Bad german proficiency | 0.83 | 0.57 – 1.20 | 0.3183 |
